# Rethinking the “Pre” in Pre-Therapy Counseling: No Benefit of Additional Visits Prior to Therapy on Adherence or Viremia in Ugandans Initiating ARVs

**DOI:** 10.1371/journal.pone.0039894

**Published:** 2012-06-26

**Authors:** Mark J. Siedner, Alexander Lankowski, Jessica E. Haberer, Annet Kembabazi, Nneka Emenyonu, Alexander C. Tsai, Conrad Muzoora, Elvin Geng, Jeffrey N. Martin, David R. Bangsberg

**Affiliations:** 1 Massachusetts General Hospital, Harvard Medical School, Boston, Massachusetts, United States of America; 2 Mbarara University of Science and Technology, Mbarara, Uganda; 3 Robert Wood Johnson Health and Society Scholars Program, Harvard University, Boston, Massachusetts, United States of America; 4 University of California, San Francisco, California, United States of America; 5 Massahusetts General Hospital Center for Global Health, Boston, Massachusetts, United States of America; 6 Ragon Institute of Massachusetts General Hospital, Massachusetts Institute of Technology, and Harvard Medical School, Boston, Massachusetts, United States of America; Rollins School of Public Health, Emory University, United States of America

## Abstract

**Background:**

Many guidelines recommend adherence counseling prior to initiating antiretrovirals (ARVs), however the additional benefit of pre-therapy counseling visits on early adherence is not known. We sought to assess for a benefit of adherence counseling visits prior to ARV initiation versus adherence counseling during the early treatment period.

**Methods:**

We performed a secondary analysis of data from a prospective cohort of HIV-infected patients in Mbarara, Uganda. Adults were enrolled upon initiation of ARVs. Our primary exposure of interest was ARV adherence counseling prior to initiating therapy (versus concurrent with initiation of therapy). Our outcomes of interest were: 1) average adherence >90% in first three months; 2) absence of treatment interruptions >72 hours in first three months; and 3) Viral load >400 copies/ml at the three month visit. We fit univariable and multivariable regression models, adjusted for predictors of ARV adherence, to estimate the association between additional pre-therapy counseling visits and our outcomes.

**Results:**

300 participants had records of counseling, of whom 231 (77%) completed visits prior to initiation of ARVs and 69 (23%) on or shortly after initiation. Median age was 33, 71% were female, and median CD4 was 133 cell/ml. Median 90-day adherence was 95%. Participants who completed pre-therapy counseling visits had longer delays from ARV eligibility to initiation (median 49 vs 14 days, p<0.01). In multivariable analyses, completing adherence counseling prior to ARV initiation was not associated with average adherence >90% (AOR 0.8, 95%CI 0.4–1.5), absence of treatment gaps (AOR 0.7, 95%CI 0.2–1.9), or HIV viremia (AOR 1.1, 95%CI 0.4–3.1).

**Conclusions:**

Completion of adherence counseling visits prior to ARV therapy was not associated with higher adherence in this cohort of HIV-infected patients in Uganda. Because mortality and loss-to-follow-up remain high in the pre-ARV period, policy makers should reconsider whether counseling can be delivered with ARV initiation, especially in patients with advanced disease.

## Introduction

From 2005 through 2009, the number of people in low income countries taking HIV antiretroviral medications (ARVs) increased over 5-fold to 5.2 million [1]. Despite this, early outcomes for HIV-infected patients remain sub-optimal, primarily in the peri-ARV initiation period [2], [3], [4]. A particular weakness of HIV programs is the failure to link patients to care after establishing ARV eligibility, when approximately 1 in 3 patients are lost to follow up in sub-Saharan Africa [5], [6], [7], [8]. Causes of such failures are complex. Structural barriers such as cost and availability of transportation are commonly cited [9], [10], [11], [12].

Adherence counseling is an important component of HIV care [13]. Repeated adherence counseling visits in patients with advanced disease prior to starting therapy, however, delays ARV initiation and may contribute to the high rate of attrition prior to initiation of ARVs. Recommendations calling for delay of ARV initiation until after completion of adherence counseling are commonly found in national HIV guidelines in sub-Saharan Africa [14], [15], [16], [17], [18], [19]. We performed an analysis of the effect of pre-therapy adherence counseling versus counseling on or after initiation of ARVs on adherence and viral suppression during the first three months of therapy in a cohort of patients initiating ARVs in rural Uganda.

## Methods

### Ethics Statement

All participants provided written informed consent to participate in the study, which was reviewed by the institutional ethics review committees of Mbarara University of Science and Technology, the Ugandan National Council for Science and Technology, Massachusetts General Hospital, and the University of California, San Francisco.

### Study Procedures

Study participants were members of the Uganda AIDS Rural Treatment Outcomes study (UARTO), a prospective cohort designed to measure predictors of ARV adherence in rural Uganda. HIV-infected patients older than 17 years who are initiating ARVs at the Immune Suppression Syndrome Clinic (ISS) of Mbarara Regional Referral Hospital were eligible for enrollment. An electronic medical record documented all clinic encounters.

Participants were enrolled on the day of ARV initiation. Blood draws for CD4 count and viral load were performed at baseline and every 3 months. Medication adherence was measured with Medication Event Monitoring System (MEMS) electronic pill bottle monitors (Aardex Group, Sion, Switzerland).

### Study Measures

Pre-adherence counseling was defined as any counseling session entitled or including “ARV Counseling” which occurred prior to the date of ARV initiation. Trained counselors perform all ARV counseling visits, which are characterized by 20-minute sessions covering dosing schedule, drug toxicities, importance of adherence including prevention of resistance, and management of missed doses. Patients who received their first episode of ARV counseling on the day of ARV initiation were not considered to have had pre-adherence counseling, as they did not require extra patient visits. Counseling was measured by review of the electronic medical record for ARV counseling visits after 2007, when counseling forms were introduced in the clinical practice. For patients with no record of ARV-adherence counseling in the electronic medical record, we performed chart review to minimize the probability of misallocation bias.

We measured time delay to ARV initiation as the time from ARV eligibility to first receipt of ARVs. Eligibility for ARVs was defined as the date of first CD4 count result below 250, the threshold for initiation in Uganda during the study period. For patients who did not have a CD4 result prior to initiation, we defined time of ARV eligibility as the date of their first counseling session.

We measured adherence over 3 months because most patients in both groups received counseling around the time of initiation, and studies have found that the effect of adherence education declines with time [20]. For our outcomes of interest, we defined poor adherence using two measurements: average adherence in the first three months of therapy <90% and absence of treatment gaps >72 hours in the first three months of therapy. For a third outcome, we defined virologic suppression as a viral load <400 copies/ml at the three-month blood draw visit.

### Statistical Analyses

Demographic and clinical characteristics were summarized by presence or absence of pre-treatment adherence counseling. Comparisons of summary characteristics across the two groups were made with chi-square testing for categorical variables and non-parametric Mann-Whitney tests for continuous variables. For binary outcomes, we performed logistic regression to detect an association between the outcome and presence or absence of pre-adherence counseling. All analyses were done using univariable modeling and again with multivariable modeling adjusted for known predictors of adherence including age, sex, travel time, employment status, socioeconomic status as measured by an asset ownership index [21], year of ARV initiation, baseline CD4 count, and history of opportunistic infection. To assess the robustness of our findings to further covariate adjustment, we did additional modeling to include depression, as measured by a score of <1.75 on the Hopkins Symptoms Score Checklist [22], and alcohol use with the AUDIT-C screen [23] in the multivariable model. These variables were not in the primary model because even though they may be related to adherence [24], they are likely affected by pre-ART counseling, so we would potentially be conditioning on part of the effect of interest. We also performed chi-squared testing to measure an association between our measures of adherence and virologic suppression to confirm a relationship between the two. Data analyses were performed using Stata Version 11.2 (StataCorp, College Station, TX).

## Results

A total of 300 of 521 total participants in the UARTO study enrolled after 2007 and had records of adherence counseling in the chart or electronic database. Two hundred thirty-one participants (77%) received adherence counseling prior to the initiation of ARVs. Of the remaining 69 patients, 88% received adherence counseling on the day of initiation. The median age of the cohort was 33 years (IQR 28–40), 71% were female, and median baseline CD4 count was 133 cell/microL (IQR 93–203). Median 90-day adherence in the cohort was 95% (IQR 85–98%), and 66% had adherence greater than 90%. There were no significant differences between those who received pre-treatment counseling and those who did not in terms of gender, age, baseline CD4, socioeconomic status, time travel to clinic, employment, history of opportunistic infection, or depression (Table 1). Participants who did not receive counseling were more likely to enroll after 2008 (71% vs 45%, p<0.01), and more likely to have a positive AUDIT-C alcohol screen (30 vs 16%, p = 0.01).

**Table 1:** Baseline demographic, socioeconomic and clinical characteristics in study cohort of patients initiating ARVs in rural Uganda.

Characteristic	Completed Pre-TherapyAdeherence Counseling(n = 231)	No Pre-Therapy AdherenceCounseling(n = 69)	p-value
Any Adherence Counseling (%)	100	88.4	<0.01
Percent Female (%)	73.2	62.3	0.08
Age (median, IQR)	33 (27–39)	33 (30–40)	0.29
Baseline CD4 (%)			0.26
<100	31.2	23.2	
100–249	54.1	65.2	
≥250	14.6	11.6	
Period of ARV Initiation (%)			<0.01
Prior to 2008	44.6	71.0	
During or after 2008	55.4	29.0	
Asset Index Quartile			0.81
1	26.6	26.1	
2	27.1	23.2	
3	24.9	30.4	
4	21.4	20.3	
Hours in Travel to Clinic (%)			0.80
<1 hour	58.4	55.1	
1–2 hours	31.2	31.2	
>2 hours	10.4	13.0	
Unemployed at baseline (%)	32.0	20.6	0.07
Every History of OpportunisticInfection (%)	41.7	45.6	0.57
AUDIT-C Alcohol Use Screen Positive	15.6	29.9	0.01
Hopkins Symptoms ChecklistDepression Score >1.75 (%)	28.7	33.8	0.41
Days from ARV Eligibility toInitiation (median, IQR)	49 (27–83)	14 (0–75)	<0.01
Days from ARV Eligibility to Initiationif CD4<100 (median, IQR)	41 (27–69); n = 72	21 (0–50); n = 16	0.04
Average ARV Adherence first3 months of Therapy	94.8	95.6	0.81
Average ARV Adherence >90%in first 3 months of Therapy (%)	64.3	72.1	0.26
Any ARV Treatment Gaps >72 hoursin first 3 months of Therapy (%)	11.7	7.3	0.29
Viral Load >400 copies/ml at3 month Follow-up Visit (%)	9.7	9.5	0.97

Participants who completed adherence counseling visits prior to therapy had significantly longer delays from ARV eligibility to initiation (median 49 vs 14 days, p<0.01). This was also true in the subset of patients with baseline CD4 counts <100 cells (median 41 vs. 21 days, p = 0.04).

In univariable analyses, completing pre-therapy adherence counseling was not associated with average adherence >90% (OR 0.7, 95%CI 0.4–1.3), absence of treatment gaps >72 hours (OR 0.6, 95% CI 0.2–1.6), or HIV viremia (OR 1.0, 95%CI 0.4–2.7). The lack of association for these outcomes persisted in multivariable models adjusted for other predictors of adherence (Table 2). Addition of alcohol use and depression as cofactors did not affect the associations. In the entire cohort, participants with treatment gaps >72 hours were more likely to have HIV viral load >400 copies/ml at 3 months (23% vs 8%, p  = 0.01). There was a non-significant increase in viremia for those with average adherence less than 90% (13 vs 7%, p = 0.12).

**Table 2:** Univariable and multivariable models for association between completion of pre-therapy adherence counseling and measures of medication adherence and persistent viremia during the first three months of ARVs in a cohort of HIV-positive patients in rural Uganda.

	Univariable Analyses		Multivariable Analyses*	
Adherence Measure	Measure of Association†	95% CI	Measure of Association†	95% CI
Average Adherence >90%	OR = 0.69	0.37–1.31	AOR = 0.78	0.40–1.54
Absence of treatment gaps >72 hours	OR = 0.59	0.22–1.60	AOR = 0.67	0.23–1.91
Persistnet Viremia >400 copies/ml	OR = 1.01	0.39–2.66	AOR = 1.13	0.41–3.12

† OR: odds ratio for odds of outcome if completed pre-ARV counseling vs no pre-therapy counseling. AOR: adjusted odds ratio for odds of outcome if completed pre-therapy counseling vs no pre-therapy counseling.

* Multivariable analysis adjusted for age, sex, time travel to from clinic, asset index quartile, baseline CD4 count, year of ARV initiation and history of opportunistic infection.

## Discussion

Patients initiating ARVs in this cohort in rural Uganda achieved high levels of adherence and viral suppression regardless of whether they received additional adherence counseling visits prior to ARV initiation or adherence counseling at the time of ARV initiation. Compared to adherence counseling delivered concurrent with ARV initiation, adherence counseling prior to ARV initiation was associated with significant delays in initiation therapy. Because this was a prospective study that enrolled participants at the time of ARV initiation, lost to follow-up could not be measured in the pre-treatment period. Multiple other studies have documented high rates of mortality and losses to follow up among eligible patients who have not initiated ARVs. At the ISS Clinic in Mbarara, approximately 30% of ARV-eligible patients during the period 2007-2009 failed to initiate ARVs within one year [25]. Multiple additional studies in South Africa, Malawi, Kenya, Ethiopia, and Mozambique have shown similarly high rates of patient attrition and early mortality in the pre-treatment period [8], [26], [27], [28]. A recent systematic review of retention rates for ARV eligible patients from 14 studies across sub-Saharan Africa corroborated these findings with an estimated median rate of attrition among ARV eligible patients of 32% (range 14–84%) [29]. Despite presentation to HIV care centers, diagnosis of HIV, and meeting clinical or laboratory criteria for eligibility, a substantial proportion of patients are failing to initiate ARVs and dying at the most critical stage of HIV disease.

When asked, patients often cite transportation costs as a primary reason for difficulty engaging in care [9], [10], [11], [29], [30], [31], [32]. They are sometimes forced to choose between purchasing basic necessities and paying for transportation visits to clinic [10]. Given the costs to patients associated with each visit, efforts to minimize the number of required visits might improve retention in care. Consistent with this hypothesis, a randomized controlled trial in Uganda found a decrease in completion of HIV testing from 99 to 68% in hospitalized patients randomized to immediate versus delayed services at a return visit [33].

Most national HIV guidelines in sub-Saharan Africa recommend some form of adherence counseling prior to initiation of ARVs in eligible patients. Of the 11 countries in the region with adult HIV infection prevalence >5% and publically available treatment guidelines, six (Botswana, Lesotho, Mozambique, Namibia, Tanzania, and Uganda) state that ARVs should be withheld from patients who have not completed counseling visits [14], [15], [16], [17], [18], [19]. The benefit of adherence counseling must be balanced by the risk of patient attrition during the pre-therapy period, where the risks are highest in individuals with advanced disease.

Multiple studies have shown benefit from adherence counseling [20], [34], [35]. The most relevant study was a randomized controlled trial of mandatory pre-therapy counseling in Kenya reported by Chung *et al.* [36]. In contrast to our findings, the study documented improved rates of medication adherence and virologic suppression among patients receiving intensive versus no medication adherence counseling. A key difference between the prior study and ours was their use of no adherence counseling in the control group, whereas 88% of patients in our delayed counseling group received counseling on the day of ART initiation. Presuming counseling has benefit, this suggests that delivering counseling on the day of ARV initiation may be as beneficial as several visits before ARV initiation. Notably, Chung *et al.* reported a rate of loss to follow up or death of 12.5% prior to initiation in those receiving counseling during mandatory pre-treatment visits, compared to 4% in the control group.

While our study leverages objective adherence monitoring in a prototypical sub-Saharan African clinic serving a rural population, our sample is small, was derived from a single clinic, and was only powered to detect a large effect size of pre-therapy counseling on early treatment adherence. Though it is very unlikely that pre-treatment counseling has a large effect on early treatment adherence, further study of this intervention will be important to evaluate for potentially important modest effects. The absence of randomized adherence counseling may introduce uncontrolled confounders despite inclusion of known predictors of adherence in our multivariate regression model. A randomized control trial comparing ARV adherence counseling prior to versus concurrent with initiation of therapy would corroborate these findings.

The high level of adherence seen in this study is characteristic of many sub-Sahara African treatment settings [24], [37]. High adherence creates a ceiling effect with respect to the ability to detect an additional benefit of pre-ART counseling. However, these high levels of adherence also question the marginal benefit of pre-ART adherence counseling, especially when pre-ART counseling delays therapy in patients with advanced disease. The patients we studied received intensive adherence monitoring, which may have altered adherence via a Hawthorne effect. While the population we studied had sufficiently high adherence not to benefit from pre-treatment adherence counseling, adherence counseling may have important benefits in select populations with lower adherence [13].

Our findings suggest that counseling at the time of ARV initiation is associated with excellent adherence and that additional adherence counseling visits prior to therapy might have at most a modest additional effect on early adherence. Counseling for both adherence and other health-related issues is a crucial aspect of HIV care and should be promoted throughout the care of the patient. However adherence counseling should not delay ARV initiation in patients with advanced disease, given high rates of death and attrition in the ARV eligibility period.

## References

[1] (2010). UNAIDS: Report on the Global AIDS Epidemic.. http://www.unaids.org/GlobalReport/default.htm.

[2] Brinkhof MW, Dabis F, Myer L, Bangsberg DR, Boulle A (2008). Early loss of HIV-infected patients on potent antiretroviral therapy programmes in lower-income countries.. Bull World Health Organ.

[3] Lawn SD, Harries AD, Anglaret X, Myer L, Wood R (2008). Early mortality among adults accessing antiretroviral treatment programmes in sub-Saharan Africa.. AIDS.

[4] Amuron B, Namara G, Birungi J, Nabiryo C, Levin J (2009). Mortality and loss-to-follow-up during the pre-treatment period in an antiretroviral therapy programme under normal health service conditions in Uganda.. BMC Public Health.

[5] Bassett IV, Wang B, Chetty S, Mazibuko M, Bearnot B (2009). Loss to care and death before antiretroviral therapy in Durban, South Africa.. J Acquir Immune Defic Syndr.

[6] Losina E, Bassett IV, Giddy J, Chetty S, Regan S (2010). The "ART" of linkage: pre-treatment loss to care after HIV diagnosis at two PEPFAR sites in Durban, South Africa.. PLoS One.

[7] McGrath N, Glynn JR, Saul J, Kranzer K, Jahn A (2010). What happens to ART-eligible patients who do not start ART? Dropout between screening and ART initiation: a cohort study in Karonga, Malawi.. BMC Public Health.

[8] Bassett IV, Regan S, Chetty S, Giddy J, Uhler LM (2010). Who starts antiretroviral therapy in Durban, South Africa?… not everyone who should.. AIDS.

[9] Weiser S, Wolfe W, Bangsberg D, Thior I, Gilbert P (2003). Barriers to antiretroviral adherence for patients living with HIV infection and AIDS in Botswana.. J Acquir Immune Defic Syndr.

[10] Tuller DM, Bangsberg DR, Senkungu J, Ware NC, Emenyonu N (2010). Transportation costs impede sustained adherence and access to HAART in a clinic population in southwestern Uganda: a qualitative study.. AIDS Behav.

[11] Maskew M, MacPhail P, Menezes C, Rubel D (2007). Lost to follow up: contributing factors and challenges in South African patients on antiretroviral therapy.. S Afr Med J.

[12] Geng EH, Bangsberg DR, Musinguzi N, Emenyonu N, Bwana MB (2010). Understanding reasons for and outcomes of patients lost to follow-up in antiretroviral therapy programs in Africa through a sampling-based approach.. J Acquir Immune Defic Syndr.

[13] Simoni JM, Pearson CR, Pantalone DW, Marks G, Crepaz N (2006). Efficacy of interventions in improving highly active antiretroviral therapy adherence and HIV-1 RNA viral load. A meta-analytic review of randomized controlled trials.. J Acquir Immune Defic Syndr.

[14] (2009). National Antiretroviral Treatment Guidelines for Adults, Adolescents, and Children. Ministry of Health Republic of Uganda.. http://www.idi.ac.ug/docs/guidelines%202009.pdf.

[15] (2008). National Guidelines for the Management of HIV and AIDS. Ministry of Health and Social Welfare, United Republic of Tanzania.. http://www.fhi.org/NR/rdonlyres/.

[16] (2010). National Guidelines for HIV & AIDS Care and Treatment. Ministry of Health and Social Welfare, Government of Lesotho.. 3rd Edition ed: Ministry of Health and Social Welfare, Government of Lesotho..

[17] (2010). National Guidelines for Antiretroviral Therapy. Ministry of Health and Social Services, Directorate of Special Programmes, Republic of Namibia.. http://www.healthnet.org.na/documents/hiv_aids/National%20Guideline%20for%20Antiretroviral%20Therapy-%203rd%20Edition.pdf.

[18] (2006). Tratament Antiretroviral e Infeccoes Oportunistas Adulto e Adolescente. Ministerio de Saude, Mozambique.. http://www.aidstar-one.com/sites/default/files/treatment_documents/hiv_treatment_guidelines_mozambique_2006.pdf.

[19] (2008). Botswana National HIV/AIDS Treatment Guidelines: 2008 Version. Department of HIV/AIDS Prevention and Care, Botswana Ministry of Health.. http://www.moh.gov.bw/templates/moh/File/BOTSWANA%20HIVAIDS%20TREATMENT%20%20GUIDELINES%20%28November%201%202008%29.pdf.

[20] Goujard C, Bernard N, Sohier N, Peyramond D, Lancon F (2003). Impact of a patient education program on adherence to HIV medication: a randomized clinical trial.. J Acquir Immune Defic Syndr.

[21] Filmer D, Pritchett LH (2001). Estimating wealth effects without expenditure data–or tears: an application to educational enrollments in states of India.. Demography.

[22] Derogatis LR, Lipman RS, Rickels K, Uhlenhuth EH, Covi L (1974). The Hopkins Symptom Checklist (HSCL): A self-report symptom inventory.. Behavioral Science.

[23] Bush K, Kivlahan DR, McDonell MB, Fihn SD, Bradley KA (1998). The AUDIT alcohol consumption questions (AUDIT-C): an effective brief screening test for problem drinking. Ambulatory Care Quality Improvement Project (ACQUIP). Alcohol Use Disorders Identification Test.. Arch Intern Med.

[24] Mills EJ, Nachega JB, Bangsberg DR, Singh S, Rachlis B (2006). Adherence to HAART: a systematic review of developed and developing nation patient-reported barriers and facilitators.. PLoS Med.

[25] Geng E, Muyindike W, Glidden D, Bwana M, Yiannoustos C, et al. Failure to Initiate ART, Loss to Follow-up and Mortality among HIV-infected Patients during the pre-ART Period in Uganda: Understanding Engagement in Care in Resource-Limited Settings. Paper 1017.; 2011 February 27 - March 3.. ; Boston, USA.

[26] Zachariah R, Tayler-Smith K, Manzi M, Massaquoi M, Mwagomba B (2011). Retention and attrition during the preparation phase and after start of antiretroviral treatment in Thyolo, Malawi, and Kibera, Kenya: implications for programmes?. Trans R Soc Trop Med Hyg.

[27] Mulissa Z, Jerene D, Lindtjorn B (2010). Patients present earlier and survival has improved, but pre-ART attrition is high in a six-year HIV cohort data from Ethiopia.. PLoS One.

[28] Micek MA, Gimbel-Sherr K, Baptista AJ, Matediana E, Montoya P (2009). Loss to follow-up of adults in public HIV care systems in central Mozambique: identifying obstacles to treatment.. J Acquir Immune Defic Syndr.

[29] Rosen S, Fox MP (2011). Retention in HIV care between testing and treatment in sub-Saharan Africa: a systematic review.. PLoS Med.

[30] Geng E, Bangsberg D, Musinguzi N, Emenyonu N, Bwana M (2009). Understanding Reasons for and Outcomes of Patients Lost to Follow-Up in Antiretroviral Threrapy Programs in Africa Through a Sampling-Based Approach.. J Acquir Immune Defic Syndr.

[31] Mshana GH, Wamoyi J, Busza J, Zaba B, Changalucha J (2006). Barriers to accessing antiretroviral therapy in Kisesa, Tanzania: a qualitative study of early rural referrals to the national program.. AIDS Patient Care STDS.

[32] Hardon AP, Akurut D, Comoro C, Ekezie C, Irunde HF (2007). Hunger, waiting time and transport costs: time to confront challenges to ART adherence in Africa.. AIDS Care.

[33] Wanyenze RK, Hahn JA, Liechty CA, Ragland K, Ronald A (2011). Linkage to HIV care and survival following inpatient HIV counseling and testing.. AIDS Behav.

[34] Pradier C, Bentz L, Spire B, Tourette-Turgis C, Morin M (2003). Efficacy of an educational and counseling intervention on adherence to highly active antiretroviral therapy: French prospective controlled study.. HIV Clin Trials.

[35] Tuldra A, Fumaz CR, Ferrer MJ, Bayes R, Arno A (2000). Prospective randomized two-Arm controlled study to determine the efficacy of a specific intervention to improve long-term adherence to highly active antiretroviral therapy.. J Acquir Immune Defic Syndr.

[36] Chung MH, Richardson BA, Tapia K, Benki-Nugent S, Kiarie JN (2011). A randomized controlled trial comparing the effects of counseling and alarm device on HAART adherence and virologic outcomes.. PLoS Med.

[37] Oyugi JH, Byakika-Tusiime J, Charlebois ED, Kityo C, Mugerwa R (2004). Multiple validated measures of adherence indicate high levels of adherence to generic HIV antiretroviral therapy in a resource-limited setting.. J Acquir Immune Defic Syndr.

